# Nomenclature

**Published:** 1895-12

**Authors:** Grant Molyneaux

**Affiliations:** Cincinnati, O.


### Nomenclature.

BY GRANT MOLYNEAUX, D.D.S., CINCINNATI, O.

Abstract of report read at American Dental Association, Aug. 1895.

The report that I have is supplemental to Dr. Guilford's report, and is in the same line, but pertaining to prosthesis.

The department of prosthesis differs in its nomenclature from the other departments of dentistry in the fact that it contains a great many common words to which we have given a distinctive meaning, and to which the definitions, as found in the dictionaries, would not be applicable. For example, the words "cast" and "model" are often used by dentists as synonyms. The former is found in one dictionary only. "Model" is not recognized in any of the dictionaries to which I have had access.

The accepted meaning of "model" is, "a mechanical imitation or copy of an object; something to be patterned after; anything of a particular form or shape to be imitated."

The word "cast" is given a special meaning in only one dictionary. In all others it is given the general meaning, as "anything formed in a mold; a mass of plastic material which has taken the shape or form of some cavity."

Many dentists use these words interchangeably, while others give them a distinctive meaning, as follows:

A "plastercast" is a fac-simile of a mouth, in plaster of Paris, upon which a denture of celluloid or vulcanite base is to be molded; a plaster cast for vulcanite; a tin cast for molding celluloid; a marble dust and plaster cast for Watt's metal.

When the plaster cast is to be used for making a mold for dental dies to be used for swaged work, it is then called a "model," because it is then to be imitated in metal, and then only is it to be called a "model."

This certainly seems to be a very excellent method, for, by using these terms with a distinctive meaning, description is abbreviated by doing away with explanatory sentences. When we refer to a "cast," we know exactly what it is. When we refer to a "model," we mean that it is to be imitated in metal.

"Vulcanite" and "rubber" are often used as synonyms, the former certainly being the proper term. Caoutchouc or rubber is a definite hydro-carbon. When this substance is combined with sulphur and other ingredients and is vulcanized, or hardened, by subjecting it to a definite temperature for a definite time, it forms a different compound, which in no manner resembles rubber, but is an ivory-like substance which we call vulcanite.

"Velum" and "obturator" are used by many interchangeably. It seems to the committee, however, that such terms can be given a distinctive meaning, even if the function of these appliances be one and the same. By applying "obturator" to vulcanite appliances used in the treatment of cleft palate, and "velum" to flexible vulcanite, the terms seem to be sufficiently suggestive to be retained.

In the metallurgical department we have many terms used improperly, such as "white metal," "platinized silver," "platinized gold." It is not infrequent to hear the term "platinized gold" used when referring to gold alloyed with platinum, often called "clasp metal" and "spring metal."

The most modern information on this subject says: "A metal *covered* by platinum is platinized; preferably by electrolysis."

I will refer to the definitions of the Century and Standard dictionaries: "Platinize: to coat with platinum in a fine state of division; platinized, by dipping in a solution of platinum chloride and then heating in a closed chamber until the metal decomposes."

According to the Standard: "Platinize: to coat with platinum, preferably by electrolysis."

In order to get an idea of this matter from a goldsmith, I sent for a piece of platinized gold of definite gauge, and received a piece of metal, one side being gold and the other platinum, equal weights of the two metals, welded so as to be inseparable.

We have the same combination in the supply-houses, known as "platinized gold" or "crown gold," "platinum" and "gold crown-metal."

The best authorities say "platinous" is the proper word, as "platinous silver," silver *containing* a small percentage of platinum; "platinous gold," gold *containing* a small percentage of platinum.

It also seems that "platinous silver" is to be preferred to the term "white metal," as the latter term is vague and inexpressive, for there are many combinations of nickel known as "white metal."

By adhering to the use of "platinous alloy," "platinous silver," "platinous gold," we have a term that means exactly what it says. It is strictly in line with chemical nomenclature, and is susceptible of improvement. We have now one combination of platinum and gold which we know as "clasp metal." We call it "platinous gold." If for any reason the proportion of platinum in an alloy needs to be increased, we can then have a platinic alloy. If we need to have more compounds, the word is susceptible of improvement by the prefixes "hyper" and "hypo," so that we can always designate the compound by a definite term. While it does not seem that these improvements are necessary, the word "platinous" is the proper one, according to all authorities, and should be adopted when referring to clasp metal or spring gold containing a small amount of platinum, instead of the term "platinized gold."

There is at the present time a good deal of discussion as to the words "aluminum" and "aluminium." A rule has obtained for fifty years or more which designates the rarer metals by an extra "i," such as "palladium," "osmium," "rubidium." When the metal comes into common use, the extra "i" is dropped, as in the case of aluminum. This would seem to be an excellent rule to follow, because it distinguishes the rare metal from the metal in common use.

Dr. Black has suggested substituting the name "occluding frame" for the word "articulator," in order to be in harmony with the nomenclature of the dental anatomy. Your committee suggest the continuation of the term "articulator:" First, because of its long usage; second, because it is a single word; third, because it is a word more suggestive of the operation to be performed, and we cannot see that it will in any manner interfere with the nomenclature of the dental anatomy.

"Autogenous soldering" is a term often applied to the process which we know as "sweating." The best authorities tell us that it is used erroneously in this sense. It is not quite decided what word would be best, "welding," "sweating," or whether to adopt the term "autogenous soldering." It is still under consideration.

The words "prosthesis" and "prothesis" are used as synonyms. The meaning of the prefixes being slightly different in the original language, "pro" meaning "to put before or to stand before;" "pros" meaning "to place, or to put to place or position," the committee has adopted the word "prosthesis" as being the proper term to use.

